# Return to Everyday Activity in the Community and Home: a feasibility study for a lifestyle intervention to sit less, move more, and be strong

**DOI:** 10.1186/s40814-019-0467-9

**Published:** 2019-06-28

**Authors:** Maureen C. Ashe, Nicola Y. Edwards, Amanda Taylor, Laura Burnett, Lora Giangregorio, Kate Milne, Lindy Clemson, Lena Fleig

**Affiliations:** 1 Department of Family Practice, Centre for Hip Health and Mobility, 7F–2635 Laurel Street, Vancouver, BC V5Z 1M9 Canada; 2 The University of British Columbia, Vancouver, Canada; 3 The University of Adelaide, Adelaide, Australia; 4 University of Waterloo, Waterloo, Canada; 5 Cardea Health Consulting, Vancouver, Canada; 6 University of Sydney, Sydney, Australia; 7 MSB Medical School Berlin, Berlin, Germany; 8 Schlegel Research Institute for Aging, Waterloo, Ontario Canada

**Keywords:** Balance and strength, Dual-process theory of behavior change, Habit strength, Physical activity identity

## Abstract

**Background:**

Many interventions designed to meet physical activity guideline recommendations focus on a single component (e.g., walking), to the detriment of other elements of a healthy lifestyle, such as reducing prolonged sitting and doing balance and strength exercises (i.e., bundled multiple behaviors). Adopting these multiple health behaviors within daily life routines may facilitate uptake and support longer-term behavior change. We tested feasibility for a three-part lifestyle intervention to support older women to sit less, move more, and complete balance and strength exercises.

**Methods:**

We used a convergent parallel mixed-methods, single-arm study design to test feasibility for a 6-week lifestyle intervention: Return to Everyday Activities in the Community and Home (REACH). We collected information at baseline, 3 and 6 weeks (final), and 6 months (follow-up) using questionnaires, semi-structured interviews, and performance-based measures. We describe three key elements: (1) implementation factors such as recruitment, retention, program delivery, and adherence; (2) participants’ acceptability and experience with the program; and (3) health outcomes, including participants’ global mobility, activity, and perceptions of their physical activity identity, and habit strength for (i) physical activity, (ii) breaking up sitting time, and (iii) balance and strength exercises.

**Results:**

We were able to recruit enough participants in the allotted time to conduct one cycle of the REACH group-based program. There were 10 community-dwelling women, median (p25, p75) age 61 (57.5, 71) years, who completed the study. The program was feasible to deliver, with high attendance (mean 5/6 sessions) and positive overall ratings (8/10). Participants rated session content and length high, and educational materials as highly acceptable and understandable. Although participants were active walkers at baseline, few were breaking up prolonged sitting or participating in any balance and strength exercises. At final and follow-up assessments, participants reported developing habits for all three health behaviors, without diminishing physical activity.

**Conclusion:**

These results show acceptability of the program and its materials, and feasibility for bundling multiple health behaviors within the REACH program. It also provides confirmation to advance to testing feasibility of this three-part lifestyle intervention with older, less active, adults.

**Trial registration:**

ClinicalTrials.gov Identifier, NCT02786394; May 18, 2016.

## Background

The effect of physical activity on chronic disease prevention throughout the lifespan is well documented [1] and is particularly important in later life, when almost half of the burden of disease in high-income countries is attributable to adults 60 years and older [2]. Engaging in healthy behaviors, such as physical activity, is one of the simplest and most effective methods of preventing or managing chronic illness and reducing mortality risk [1, 3]. Despite the available evidence and guideline recommendations, at a population-level, few older adults participate in sufficient physical activity [4] and are thus considered “inactive” (i.e., do not meet physical activity guidelines) [5, 6].

Based on a systematic review, < 18% of older adults (across many countries) meet activity guidelines for moderate-to-vigorous physical activity (MVPA) collected via accelerometry [7]. Furthermore, there are health risks associated with prolonged sedentary behavior (distinct from being inactive) [8, 9], and older adults spend the most time sedentary of all age groups [8, 10]. Guidelines recommend that older adults perform balance and strength exercises, key in preventing falls [11], but only 16% of older adults from a US population-level survey met strength training recommendations (at least twice a week), and this number is lower if the person has a mobility limitation [12]. As a final point, based on US population-level data, few adults 65 years and over meet both MVPA and strength training recommendations [13]. However, to support health and aging, it is important to address these three health behaviors concurrently: physical activity (move more), reduce sedentary behavior (sit less), and perform balance and strength exercises (be strong).

Psychosocial factors influence activity behaviors [14, 15], and despite the best plans, there is a known gap between physical activity intentions and behavior [16]. Balance and strength exercises, for example, may be intimidating or perceived as unrealistic for many older adults [17]. Interventions based on behavior change theory and techniques may mitigate some of the obstacles. The 2018 US physical activity guidelines note the effectiveness for individualized programs, based on behavior change theory and techniques, to increase the volume of physical activity in adults [18]. Evidence suggests that it is feasible [15] and effective [19] to anchor new activities around existing lifestyle routines. For instance, completing small knee bends (target behavior) at the kitchen sink while doing the dishes (i.e., activity-based cue to action: doing the dishes is a reminder to complete the exercise) [14, 20]. Theory-based principles of habit formation [21] that guide lifestyle interventions can encourage older adults to engage in small incremental changes of physical activity to foster self-efficacy via mastery [22] and longer-term behavior change and/or maintenance [23]. Emerging evidence highlights the health benefits of light physical activity [24–26], and this is supported by the recently updated US physical activity guidelines [18]. Although more intense physical activity (MVPA) may yield better health benefits [1, 27], lower energy activities can serve as a foundation (or building block) to gradually enable higher intensity activity [28].

Challenges exist to adopt health behaviors in general, and in particular, when introducing multiple behaviors simultaneously. “Bundling” more than one health behavior as part of an intervention may have positive or negative consequences [29]. For instance, completing one of the activities may be a gateway behavior to adopt all three activities [30–33]. Conversely, adding too many activities at once may be overwhelming [34], leading to the cessation of one or more activities. A laudable goal to enhance older adults’ overall health, and reduce the risk for injury (from falls, for example), is to develop and test interventions that support daily activity (e.g., walking, activities of daily living), but also target the reduction of prolonged sitting, and promote balance and strength exercises. To do this, it is important to understand the feasibility of introducing multiple health behaviors to maximize uptake and adherence.

Therefore, we aimed to extend our previous work with women at midlife or older [14, 35] and test feasibility for a three-part lifestyle intervention for older adults. In this development phase, we directed feasibility testing towards women later in life for several reasons. Based on population-level data, compared with older men, older women generally achieve less daily moderate physical activity [36] and are at more risk for falls [37] and low-trauma hip fractures (the most serious of low-trauma fractures) [38]. Return to Everyday Activity in the Community and Home (REACH) includes activities shown to reduce falls [20] and improve older adults’ health outcomes [35], using health behavior change theory and behavior change techniques (BCTs) [14, 39]. Our objective was to investigate feasibility of REACH, and to explore selected health outcomes. Feasibility was based on participants’ acceptability and experience, program delivery, and their uptake and adherence to the program. We wanted to determine first in a younger, more active, group if we could deliver, and if participants could integrate, three “bundled” health behaviors simultaneously. In this way, we aimed to disentangle if the intervention was feasible to deliver and be adopted (implementation factors) before we tested its feasibility with less active older adults. This contextual knowledge is an essential component to refine the person-centered intervention prior to conducting a larger study.

## Methods

### Design

We used a convergent parallel mixed-methods [40] single-arm study design to investigate feasibility for a three-part lifestyle program (increasing physical activity, reducing sedentary behavior, and completing balance and strength exercises). Knowledge of this information would support (or not) our decision to test delivery of a bundled behavioral intervention in a larger study. We examined feasibility based on elements of a published framework [41], using questionnaires, semi-structured interviews, and measurement of selected health outcomes at three times (baseline, 6 weeks, and 6 months) for (1) implementation research outcomes (program feasibility defined as recruitment and retention, and program delivery and adherence), (2) person-centered outcomes (program acceptability and experience), and (3) exploration of impact (report of health outcomes).

### Participant recruitment and setting

We worked with our research institute to send approved recruitment posters to staff and sent emails to potential participants who provided consent to be contacted about future research opportunities. We based our sample size on, feasibility to recruit participants in the allocated time, the need to optimize group size (8–10 participants) for delivering the intervention, and our previous pilot work using mixed methods [14]. We excluded participants who were unable to walk four city blocks and climb one flight of stairs or were receiving treatment for medical conditions that would prevent them from taking part in a physical activity program (e.g., high blood pressure, recent injurious fall or fracture). Participants completed the Physical Activity Readiness Questionnaire for Everyone (PAR-Q+) [42] with a registered exercise physiologist to ensure their capacity to participate. We conducted the program in Vancouver, British Columbia, and registered the study (ClinicalTrials.gov Identifier: NCT02786394; May 18, 2016; https://clinicaltrials.gov/ct2/show/NCT02786394?term=NCT02786394&rank=1). The study was approved by the university and hospital research ethics boards (H16–00670), and all participants signed a consent form prior to commencing the study.

### Procedures

#### Intervention

REACH is a lifestyle intervention for active living developed from our previous work [35] and includes elements of the Lifestyle-Integrated Functional Exercise (LiFE) program [20] designed to encourage older adults to embed balance and strength exercises into everyday activities (rather than just completing a standard set of exercises). The REACH model was designed to address three health behaviors: increase levels of physical activity, reduce prolonged sitting time, and incorporate balance and strength exercises into daily life routines. The intervention also included components relevant in the lives of older adults, because recent literature highlights the importance of a full day approach [43] to being active. Therefore, we included such topics as sleep, stress reduction, and known barriers to being physically active, such as urinary incontinence and nocturia, which are also falls risk factors [44, 45]. Our long term goal is to test the acceptability and effectiveness of REACH in less active older adults with mobility limitations and/or at risk for falls. However, prior to this, we needed to confirm that we could deliver the three behaviors in a younger, more active older adult group. Table 1 is a detailed description of REACH using the Template for Intervention Description and Replication (TIDieR) Checklist [46].

**Table 1:** Template for Intervention Description and Replication (TIDieR) Checklist for REACH Participant Program

Name	REACH—Return to Everyday Activity in the Community and Home	
Rationale	A feasibility study to test factors related to participant perceptions and program delivery. REACH, a lifestyle intervention**,** aims to address sedentary and inactive lifestyles which may lead to health concerns and a reduction in quality and length of life. REACH addresses activity levels throughout the day, by applying behavior change theory and techniques to reduce sitting, increase physical activity, and incorporate strength and balance activities into life routines.	
Materials	• Activity monitor (Fitbit One or Fitbit Zip)	• Infographic handouts at each session
	• Participant manual and presentation handouts	• Activity logs at each session
	• Quiz at the last session	• Home practice activity
	• Transit map	• Exercise instruction
Instructor resources	One instructor (exercise physiologist) led the sessions with the help of one research assistant. They received a REACH manual and slides for each session	
Procedures		
Recruitment and screening	*Recruitment:* Email, online posting, and previous study participants who expressed interest *Screening:* Research assistants conducted preliminary screening and obtained consent. The REACH instructor screened participants the Physical Activity Readiness Questionnaire (PAR-Q+; some participants needed to obtain written permission from their family physician before participating in the study) and the Life Assessment Tool (LAT).	
REACH program	We delivered the REACH program in 6 sessions (1–2 h each), following the REACH manual. *Sessions breakdown:* review of previous session, introduction to new session, group discussion and activities, new exercise and home practice activity explained (stretch and activity breaks throughout), optional group-based walk (10–30 min). At the last session, participants had the opportunity to write themselves a letter about their physical activity goals.	
Behavior change techniques (BCTs)	See Table 2 for a detailed description of BCTs used within REACH.	
Research assessments	One of two trained research assistant administered and collected the following assessments:	
	• Every session: feedback forms and physical activity tracking sheets	
	• Baseline, midpoint, final, and 6-month follow-up: semi-structured interviews (in-person or via telephone)	
	• Baseline, final, and 6-month follow-up: Short Grit Scale effort subscale, Self-Report Physical Activity Identity Scale, Habit Strength for sedentary behavior, physical activity, and balance and strength exercises	
	• Baseline and final: Timed Up and Go (TUG)	
	• Final: PEMAT-P (Patient Educational Materials Assessment–Print Materials)	
Delivery		
Providers	One exercise physiologist (with 18 years of experience) led the sessions with the help of one trained research assistant.	
Mode	The program was delivered face-to-face in a group of 8 to 10 participants.	
Location	The program took place in a multipurpose exercise room at a research center.	
Sessions	REACH occurred over 6 weeks: one session/week (1–2 h) and optional walking sessions (10–30 min). Weekly content included:	
	• Session 1—introduction to the program	
	• Session 2—making a change	
	• Session 3—from action to habit	
	• Session 4—making exercise EASY	
	• Session 5—active transportation	
	• Session 6—taking your habits home	
Tailoring	When participants could not attend the regularly scheduled sessions, if possible, we provided a one-to-one session with the instructor either before or after the regularly scheduled session. The intervention occurred between 5 and 7 PM on a weekday to accommodate participants’ work schedules.	
Modifications	Originally, there were two sessions per week: one REACH session and one optional walking session. However, due to feedback from participants and low attendance for the optional walking sessions, we decided to add the optional walking session after the REACH session (i.e., all activities on 1 day).	
Adherence		
Attendance	The research assistant recorded participants’ attendance at each session.	
Physical activity tracking sheets	We asked participants to write down and submit their physical activity data.	
Other measures	Optional adherence tools (not collected): we did not collect the home practice activities to measure adherence or weekly checklist; however, we did talk about the activities within the sessions.	

As part of REACH development for future research (but separate from this feasibility study), we created an instructors’ manual and provided an instructor training course for 3 weeks in May 2016. The training sessions were delivered face-to-face in a group setting, and three exercise physiologists (average 17 [8] years of experience working with older adults) completed the course. Each of the three sessions was 1–2 h in duration. We sought their overall impression of the program and the training sessions, for future studies.

We used theory to guide the REACH intervention, specifically, a dual-process approach incorporating conscious (i.e., motivational and volitional) processes and automatic processes (i.e., habit strength) [15]*.* In this study, we investigated physical activity identity and habit strength for three health behaviors (being physically active, breaking up sitting time, completing balance and strength exercises). We chose these variables to explore participants’ perceptions of their health behaviors related to the intervention.

At baseline, participants completed the Life Assessment Tool (LAT) [47] to understand participants’ ability and as a starting point for activity prescription. REACH was delivered in a group setting with one instructor (registered exercise physiologist with 18 years of experience), with six sessions of approximately 1–2 h per week over 6 weeks (May 2016–June 2016). The exercise physiologist was a member of the intervention development team. Each session included a presentation of new information, participatory activities (e.g., think, pair, share), and one to two new balance and strength exercises, to ensure participants were able to observe and experience the optimal form for completing the exercise. Additionally, participants were encouraged to complete home practice activities each week and balance and strength exercises. Key topics in the REACH model include the importance of participating in light and leisure-time physical activity, reducing sedentary time, fracture prevention, habit formation (via BCTs such as goal setting; see Table 2), mindfulness, sleep, nocturia and urinary continence, and active transportation. These topics were chosen based on our previous work [14, 35], the importance of approaching physical activity from a 24-h perspective [48], and the overall goal of testing the program in a larger trial (with fall prevention as an outcome of interest). We also offered participants the opportunity to attend an additional (optional) once a week walking session. We had a change in protocol as participants opted to have only one session/week: that is, the REACH group session and walking program was changed to be delivered on the same day. At the last group session, participants could choose between writing a letter to themselves about their activity goals (and we mailed it 3 months later) or to have a reminder email (to maintain new habits) sent at 3 months after the final assessment at 6 weeks.

**Table 2:** Content of REACH intervention by session based on the CALO-RE taxonomy [39]

Behavior change technique	Session 1: introduction to the program	Session 2: making a change	Session 3: from action to habit	Session 4: making exercise EASY	Session 5: active transportation	Session 6: taking your habits home
Shaping knowledge (i.e., information on antecedents of habit formation)	X	X	X	X		
Shaping knowledge (i.e., instruction on how to perform the behavior)	X	X	X	X	X	
Demonstration of behavior	X	X	X	X	X	
Feedback on behavior (i.e., form)	X	X	X	X	X	X
Behavioral practice/rehearsal	X	X	X	X	X	
Graded tasks	X	X	X	X	X	
Goal setting (behavior)		X	X	X	X	X
Review of goals			X	X	X	X
Focus on past success		X	X	X	X	X
Action planning		X	X	X	X	X
Prompt/cues	X	X	X	X	X	X
Self-monitoring of behavior	X	X	X	X	X	X
Social support (practical)	X	X	X	X	X	X
Social support (emotional)	X	X	X	X	X	X
Relapse prevention/coping planning				X	X	X
Barrier identification/problem-solving				X	X	X
Habit formation	X	X	X	X	X	X

#### Data collection

Two trained research assistants collected data either in-person, via telephone, or using a secure web application.

##### Implementation research outcomes

We recorded details related to participant recruitment, retention program delivery, and adherence to the intervention. We collected feedback questionnaires after each session on the rating of overall session, content clarity, session length, and participants’ confidence in the ability to use information and perform activities, etc. Participants also completed the Patient Education Materials Assessment Tool for Print Materials (PEMAT-P) [49] to evaluate the REACH Participant Manual. The PEMAT-P assesses educational materials based on understandability (content, word choice and style, use of numbers, organization, layout and design, and use of visual aids) and actionability (ease of acting based on the written material) [49].

##### Person-centered outcomes (program acceptability and experience)

We conducted in-person or telephone semi-structured interviews at four time points: baseline, midpoint, final, and 6 months after withdrawing the intervention. We requested the participants provide feedback on acceptability of the program and explored participants’ expectations and perceptions of the intervention.

##### Exploration of impact (report of health outcomes): questionnaires and performance-based measures

Participants completed self-report questionnaires at baseline, after the intervention (6 weeks), and 6 months after withdrawal of the program: Short Grit Scale [50] effort-subscale [51], Physical Activity Identity Scale [52, 53], and the Self-Report Behavioral Automaticity Index (SRBAI) [54]. The Short Grit Scale is an eight-item questionnaire (with five response options) to assess an individual’s “grittiness” or passion and perseverance in long-term goal attainment [55]. However, we only included the “effort” subscale, which included questions 2, 4, 7, and 8 [51]. We included the Exercise (Physical Activity) Identity Scale, originally developed by Anderson and Cychosz [53] and adapted for older adults by Strachan and colleagues [52], because it may influence the relationship between intention and behavior (being active) [56]. This scale includes nine items and asks participants to give a rating on a 7-point Likert scale (strongly disagree–strongly agree); the higher the score, the stronger the perception of identifying with physical activity. The SRBAI is a reliable four-item (and seven response options) subscale of the Self-Report Habit Index (SRHI). It assesses the level of automaticity for health behaviors (habit strength) [54]. We used the SRBAI to measure habit strength for three behaviors on a 7-point Likert scale (strongly disagree–strongly agree) for (i) physical activity, (ii) breaking up prolonged sitting time, and (iii) balance and strength exercises. We also asked participants in the semi-structured interviews about their physical activity identity, habit strength, and level and types of and satisfaction with physical activity behavior. Participants wore a Fitbit monitor (Fitbit One or Fitbit Zip; Fitbit, San Francisco, CA), and we provided weekly activity tracking sheets for them to record their steps, active minutes, and sedentary time. For accelerometry variables, we calculated median daily values based on at least 4 days of wear time. Participants also completed a Timed Up and Go test (TUG) [57] (at their usual pace) at baseline and final assessment (6 weeks) to capture their global mobility.

##### Data analyses and interpretation

We provide basic descriptive information for participants, reported activity, session ratings, and other quantitative results, using means and standard deviations (SD), or medians and 25th, 75th percentiles, where appropriate. If physical activity variables were missing at the final assessment (week 6), we imputed values from week 5. This was a feasibility study; thus, we did not conduct any inferential statistical analyses, but provide boxplots to display the results (median, range, outliers) for physical activity identity and habit strength variables. We used IBM SPSS Statistics Version 25 (IBM Armonk, New York).

##### Semi-structured interviews

We recorded and transcribed interviews verbatim and coded them using NVivo Software (QSR International Pty Ltd., Doncaster, Victoria). We were guided by Thorne’s Interpretive Description [58]. At each time point, two authors read interview transcripts multiple times and discussed initial emerging concepts. Two authors (NE, MCA) created an analysis plan after preliminary examination of the first time point interviews, and one author (NE) led the initial broad-based coding for all time points and kept a coding activity journal. We focused our interpretive description on the participants’ physical activity identity and behavior change perceptions over time. We used multiple forms of triangulation (data, investigator, and methods) to increase rigor in our analysis. We provide quotes to support findings and assigned pseudonyms to maintain participants’ anonymity.

## Results

**Fig. 1:**
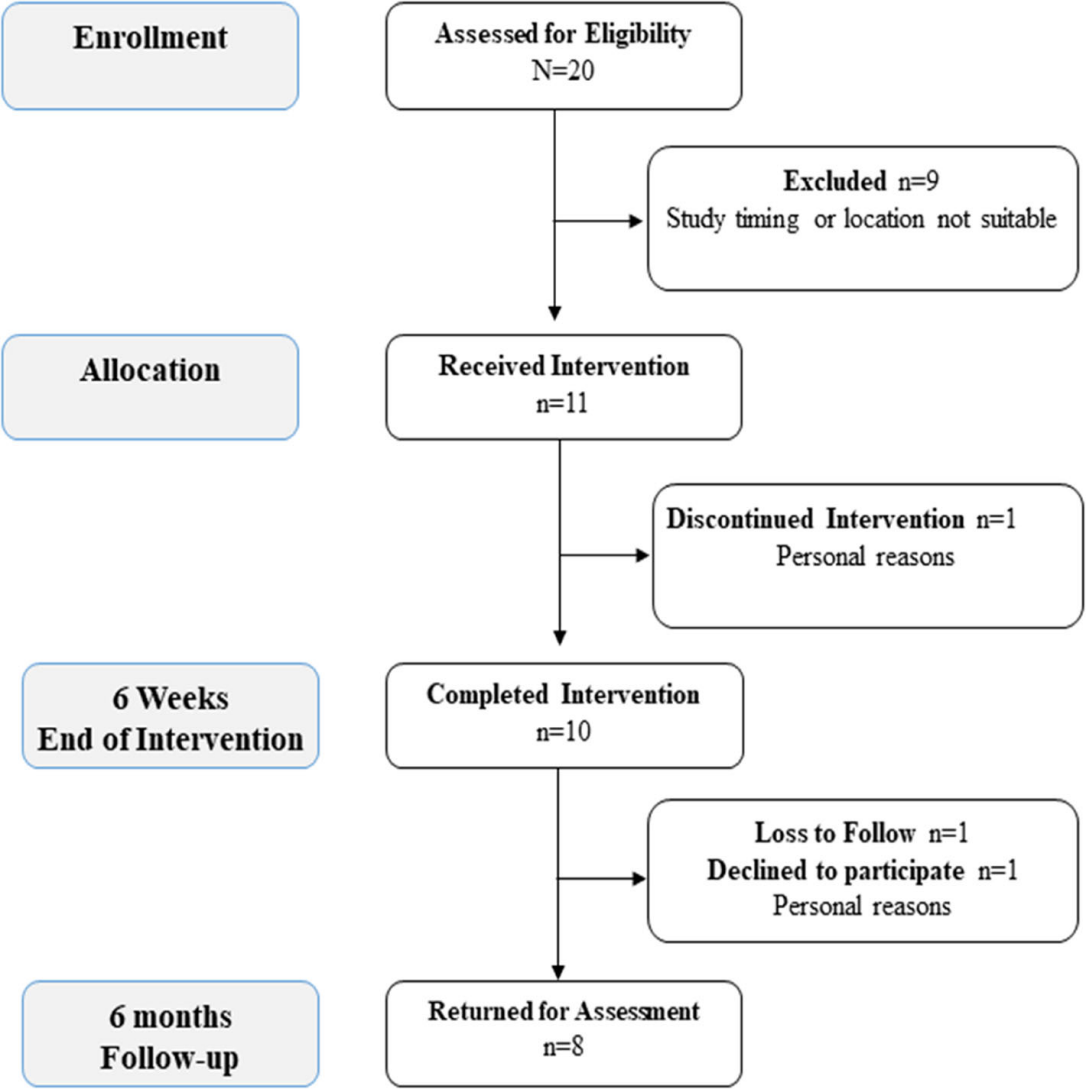
CONSORT flow diagram

The study ran from May–June 2016, with a follow-up assessment 6 months later. During the recruitment phase, 20 participants contacted us to receive more information about the study; however, nine participants declined to participate because either the timing or the venue was not convenient (Fig. 1). We enrolled 11 participants, but one participant withdrew (for personal reasons) after the second session. Ten healthy community-dwelling women between 55 and 77 years (median (p25, p75) 61 (57.5, 71)) completed the six REACH sessions and data collection at baseline, midpoint, and final (10/11 retention). Participants’ median body mass index (BMI) was 23.5 (21.4, 26.6). All participants completed high school, and nine had university education; seven women were employed, and three were retired. Generally, participants were very active, with 8343 (5308, 12,641) median steps/day at baseline. Their median TUG time was 9.1 (7.7, 9.8) s at baseline, well below the cut point for increased fall risk [57].

### Implementation research outcomes

We recruited the target number of participants in the allotted time and had excellent retention to the program (10/11 participants). At the 6-month follow-up assessment, one participant was lost to follow-up, and one declined to participate for personal reasons (8/10 retention at 6 months). We could not conduct an interview with one participant (unavailable) for the 6-month follow-up interview, but they provided data for all other measures (Fig. 1). Participants attended, on average, 5/6 sessions; this includes five make-up sessions delivered to three participants one-to-one either before or after the regularly scheduled group-based session. Participants did not regularly attend the walking sessions held on a different day; therefore, we combined the sessions and only offered one session/week. Following this, the research assistant did not take separate attendance for the walking component after the program modification. Based on the PEMAT-P evaluation, REACH participants rated the manuals 100% for actionability and 98% for understandability.

### Person-centered outcomes

Participants’ overall ratings (content clarity and method/style of teaching) and confidence in the ability to use information and perform activities were consistently above 5 on a 7-point scale and improved over the 6 sessions. Participants rated their overall experience with the program 7.9/10 at the midpoint (3 weeks) and 8.1/10 at the final point (6 weeks). Results from the semi-structured interviews indicated that participants were initially unsure what to expect with the program, and assumed the intervention would be much more physically intensive. Initially saying, “I did not know what to expect” (Sarah). However, at midpoint, participants reported positive experiences with the program that were counter to their expectations, “I’m pleasantly surprised” (Carole), and “[The program is] quite different than what I anticipated, but in a good way. It’s been an education for me versus just a go and do something which is kind of what I expected” (Brie). Overall, participants expressed gratitude for the research team tailoring the program to the participants’ preferences.

Participants’ key take away messages from REACH included personal awareness of habit cues, the importance of reducing sedentary behavior, and the benefit of small increments of activity. They appreciated the simplicity of the activities and the small amount of time commitment, making it more manageable to gain health benefits. Many participants noticed new opportunities to be active and added activity throughout their day in ways that they did not think were important, or beneficial, before the REACH program. For example, walking up the escalator, standing up while folding laundry, and or standing on one foot while waiting in line at a store. They also appreciated learning about the importance of reducing sedentary time and improving balance and strength, as this was new information for some participants. “I tended to not care whether I got up from my desk at all for the whole day. And then just did my walk at night or something like that. I don’t do that anymore. I’m much more mindful of getting up, even if it’s just a little bit every hour” (Brie). They also discussed observing small declines in their balance in recent years, and how this triggered concern for some, and acted as motivation for others. Susan explained, “when you get old you don’t realize how your perceptions of things change…. and then some little thing will happen, and you start to realize how easy it would be to fall and hurt yourself, and so how important it is to get your strength and balance up”.

Despite the successes associated with the program, we noted two challenges. First, we planned to deliver two sessions per week: the group-based REACH session and one optional walking session (on a separate day). However, due to low attendance for the optional walking sessions (on a separate day), we consulted with participants, and the walking session was rescheduled after the REACH session (i.e., only one session/week). Second, to collect physical activity and sedentary behavior data, we provided Fitbits to some participants, or they used their own. As this was a feasibility study, we requested the participants submit written sheets with Fitbit generated data. However, there were some missing sheets, and only step count data were recorded and available for analysis.

### Exploration of impact (report of health measures): questionnaires and performance-based measures

**Fig. 2:**
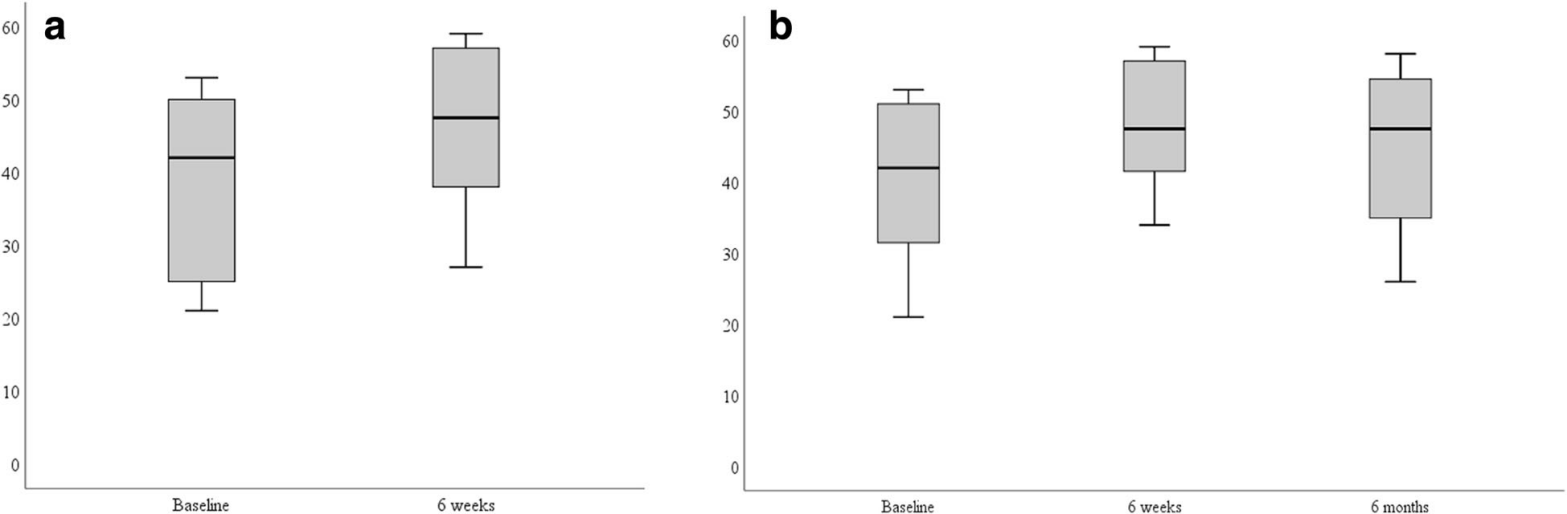
Boxplots for physical activity identity. **a** Data from 10 participants and **b** data from the eight participants who provided data at baseline, final, and follow-up (6 months) for physical activity identity. The total possible score is 63 points, and a higher number indicates a stronger identity with physical activity

**Fig. 3:**
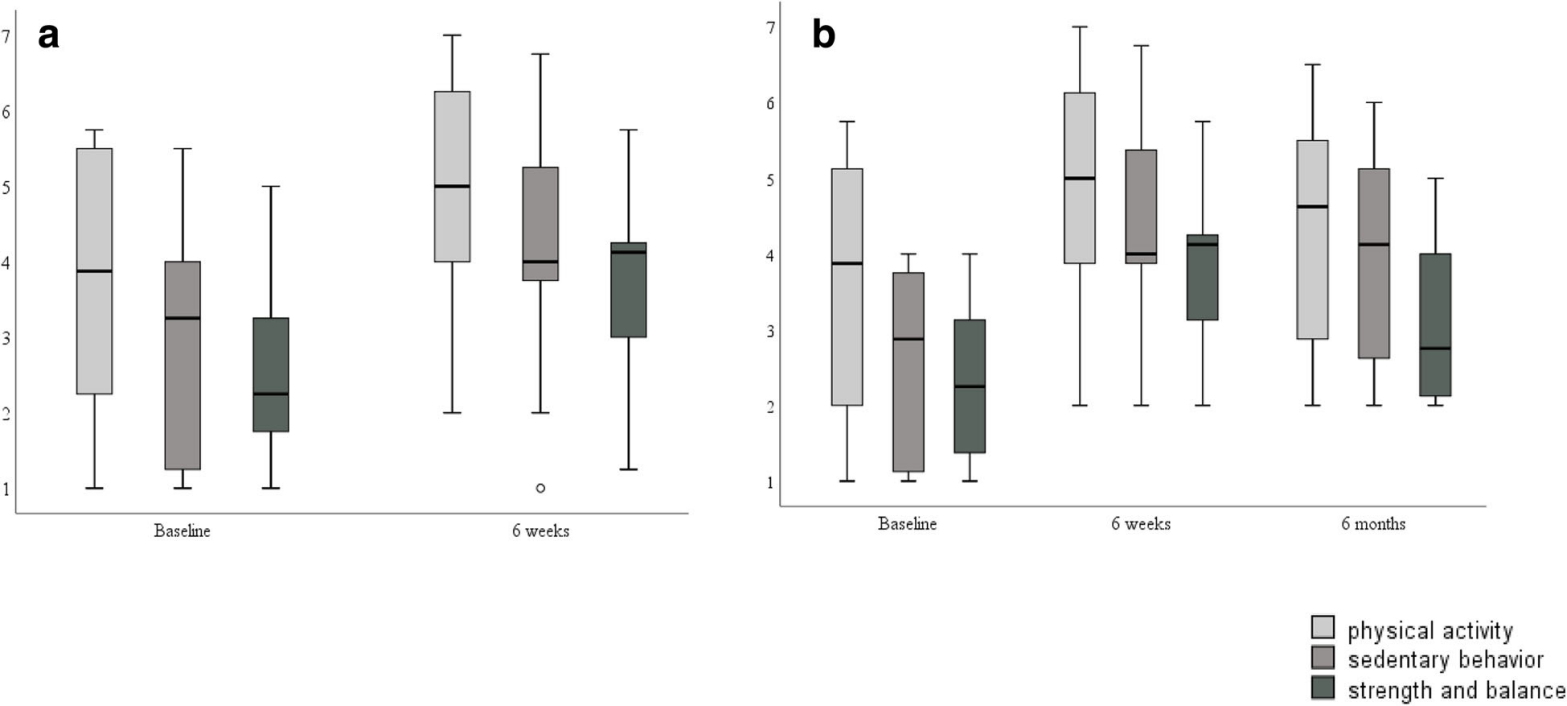
Boxplots for habit strength for physical activity, (breaking up) sedentary behavior, and balance and strength exercises. **a** Data from 10 participants and **b** data from the eight participants who provided data at baseline, final, and follow-up (6 months) for habit strength for the three activities. Scores are based on a 7-point Likert scale (strongly disagree–strongly agree)

Figures 2 and 3 respectively are boxplots for physical activity identity and the habit strength (physical activity, breaking up sedentary behavior, and balance and strength) measures. We present data for the 10 participants who completed the study (6 weeks) and the eight participants who completed the study and returned 6 months later. In the boxplots, we note the variability in responses across measures and time; there was only one data point considered an outlier for sedentary behavior habit strength at 6 weeks (*n* = 10). However, the median values increased for physical activity identity and habit strength from baseline to 6 weeks. We provide a summary of the other measures across time points in Table 3.

**Table 3:** Participant data (median (25th, 75th percentiles)) at baseline, final, and 6 months follow-up

Characteristic	Baseline	Final	6 months follow-up
Short Grit Scale effort subscale, x/5	3.9 (3.2, 4.6)	4.1 (3.4, 4.5)	3.6 (3.1, 4.3)
Steps/day	8343 (5308, 12,641)^(*n* = 9)^	7954 (6861, 11,401)^(*n* = 8)^	–
Timed Up and Go, s	9.1 (7.7, 9.8)	8.4 (7.7, 9.2)	–

At baseline and final, there were 10 participants, but only eight participants at the 6-month follow-up data collection period

### Semi-structured interviews: physical activity identity

At each interview, we asked participants if they self-identified themselves as a physically active person. Many struggled to answer this question directly and often provided an explanation or justification for their response. For example, Susan responded, “I don’t see myself as being as physically active as I would like to be.” Similarly, Carole said, “I don’t see myself as physically active… But I am active. I mean, I walk to work and stuff. So …Other people’s terms, yes. In mine, no.” Many also recalled being active in the past, but not anymore, Patricia said, “I used to be very physical when I was younger but, you know, when you start getting problems you stop doing things.” Six months after withdrawing the intervention, 5/7 participants answered positively about perceiving themselves as physically active, some explaining, “yes, you know, relative to my age,” and one explaining “yes, more than before.”

### Habit strength

Data from the semi-structured interviews highlighted that participants endorsed the concept of linking new behaviors with routine-based cues (i.e., habit formation). They shared personal strategies that helped them change or develop new health behaviors. Participants spoke of looking for opportunities to walk more as an alternative mode of transport or adding in walks, even if only 10 min in duration. One participant mentioned she brought sneakers to work, “...so that if there’s any opportunity I can walk, I have comfortable shoes” (Sarah)*.* Many mentioned performing the balance and strength exercises in their regular routines in situations where they had to wait. Beth explained, “when I’m waiting for something to scan or photocopy or, you know, I’m going to talk to someone, sometimes I do the toe thing (a balance activity).” Some found it easier to develop cue associations in the workplace while others did at home. Patricia found home routines, such as “watching some TV, during the break and doing the exercises then [or] …brushing teeth or…standing there waiting for the water to boil for tea or… getting things done in [the] kitchen,” were valuable opportunities to link with balance and strength exercises. Six months after withdrawing the intervention, 5/7 participants believed their involvement in REACH helped them change or develop new health behaviors or habits, and 6/7 participants reported regularly breaking up their sedentary time.

### Physical activity

Six of the seven (86%) who participated in the follow-up interviews reported participating in physical activity programs (either self-directed or group-based regular activity) since the REACH program. At the 6-month follow-up interview, we asked participants about their satisfaction with their level of physical activity. Although many of the participants believed that they had changed their health behaviors, and would identify as physically active, they were generally not very satisfied with their current level of physical activity. Many believed they should increase their activity levels and frequency. Only two participants were satisfied with their level of physical activity, although one of these participants mentioned she wanted to incorporate more balance activities.

## Discussion

This study highlights feasibility for delivering an intervention with “bundled” health behaviors to facilitate an increase in everyday activity (including walking), reduction in sedentary behavior, and adoption of balance and strength exercises. Participants’ overall impression of the REACH program was very positive. Session content and length were rated high, and educational materials were highly acceptable and understandable. Although participants were active walkers at baseline, few were breaking up prolonged sitting or participating in any balance and strength exercises—nor were they aware of the importance of these health behaviors. However, at final and follow-up assessments, participants reported developing habits for all three health behaviors, without diminishing their physical activity. That is, participants maintained their high level of daily step count at 6 weeks, even with the addition of two new behaviors. An interesting finding emerged from the data. Within the semi-structured interviews, many participants stated they did not perceive themselves as “active” or were satisfied with their activity levels, despite high levels of physical activity behavior (daily step count). Overall, our findings support feasibility to deliver the program as intended, participants reported acceptability of REACH program and materials, and reported adopting and maintaining habits for the three target activities.

Participants’ reported developing habits for breaking up prolonged sitting and engaging in balance and strength while their physical activity behavior remained high (median ~ 8000 steps/day) from baseline to final. With the addition of the two new behaviors, participants’ activity was more aligned with recommended guidelines for health [18]. Adopting these three “bundled” behaviors throughout the day is important (and thus the reason for their inclusion in public health messages); however, it is also easy to let one or more activities drop off over time. It is not surprising that walking is the most popular form of exercise for older adults [4], but especially with aging, it is essential to incorporate all three activities into daily life routines. It is important for adults, and particularly older adults, to routinely engage in balance and strength exercises [11]. It is an essential component of the physical activity guidelines but frequently is in the shadow of MVPA when discussing who is sufficiently active. The most recent Cochrane Review of fall prevention and exercise by Sherrington and colleagues reported there was high-certainty evidence for balance and functional exercises to reduce the rate and number of people who fall, and moderate-certainty evidence for a combined program (balance and functional exercises plus resistance training) for the same outcomes. However, the review stated there was not enough evidence that walking alone reduced fall-related outcomes for this population [59]. There is a possible increased risk for falls with higher volumes of physical activity (outdoor walking) [60], and reducing prolonged sedentary behavior is an important goal especially as recent evidence showed sitting > 8 h/day was associated with an increased risk of falls [61].

We observed an unexpected finding in this study. Participants were active walkers (median > 8000 steps/day), and their Physical Activity Identity Scale scores were within the range of “exerciser” (40.6 to 45.6) [62]. Yet, in the semi-structured interviews, several women commented that even though they knew they were active, they were not satisfied with their activity levels. We do not know the reason for the discordant findings between data from interviews and questionnaire, but it opens up several areas for future investigation. First, it is possible that just because someone identifies with physical activity, does not always equate with satisfaction with current physical activity levels. Second, there is evidence from younger women that there may be difficulties identifying physical activity intensity [63]. That is, women were engaging in vigorous activity, but perceived it as moderate exercise [63]. Thus, our participants may have experienced a similar misperception of their exercise level. However, as neither the questionnaires nor the interview questions introduced a distinct definition of physical activity, it remains unclear to which type and intensity of physical activity participants referenced when answering the items and questions. Finally, physical activities of lower intensity levels (e.g., reducing sitting and balance, and strength exercises) may not necessarily contribute to perceptions of activity. However, this finding identifies an area to explore in our future studies.

We acknowledge several limitations with this study. For example, we recognize the limited generalizability due to the small sample size. Additionally, the participants were relatively active and may not represent women for this age group. Our measures were limited primarily to self-report data, and we did not collect extensive physical function tests, as we did not expect a significant change in the short time period. Future studies should include other psychosocial measures in addition to identity and habit strength which are key to the dual-process model, such as action planning and intention and outcome expectancies. Many of these concepts were taught in the intervention; however, we did not examine them in the current study.

## Conclusion

Overall, we were able to recruit enough participants in the allocated time and had a high retention of participants to the 6-week intervention and at 6 months follow-up. Participants described positive experiences with the REACH intervention, materials, and delivery. They stated the intervention was simple and manageable and reported high confidence in their ability to use the information and perform the new activities. Participants reported developing habits for all three activities (sitting less, moving more, and performing balance and strength exercises), without diminishing their overall physical activity. Collectively, these findings guide the next phase of testing the feasibility of this intervention in an older, less active, population, and suggest future areas of investigation into the psychosocial factors that support adoption and maintenance of positive health behaviors.

## Data Availability

The datasets used and/or analyzed during the current study are not available.

## Abbreviations

BCT: Behavior change techniques
LAT: Life Assessment Tool
LiFE: Lifestyle-Integrated Functional Exercise
MVPA: Moderate-to-vigorous physical activity
PAR-Q+: Physical Activity Readiness Questionnaire for Everyone
PEMAT-P: Patient Educational Materials Assessment-Paper
REACH: Return to Everyday Activity in the Community and Home
SRBAI: Self-Report Behavioral Automaticity Index
SRHI: Self-Report Habit Index
TIDieR: Template for Intervention Description and Replication
TUG: Timed Up and Go test

## References

[1] Warburton DE, Nicol CW, Bredin SS (2006). Health benefits of physical activity: the evidence. CMAJ : Canadian Medical Association journal = journal de l'Association medicale canadienne.

[2] Prince MJ, Wu F, Guo Y, Gutierrez Robledo LM, O'Donnell M, Sullivan R (2015). The burden of disease in older people and implications for health policy and practice. Lancet..

[3] Bouaziz W, Vogel T, Schmitt E, Kaltenbach G, Geny B, Lang PO (2017). Health benefits of aerobic training programs in adults aged 70 and over: a systematic review. Arch Gerontol Geriatr.

[4] Ashe MC, Miller WC, Eng JJ, Noreau L, Physical A (2009). Chronic conditions research T. older adults, chronic disease and leisure-time physical activity. Gerontology..

[5] Tremblay MS, Aubert S, Barnes JD, Saunders TJ, Carson V, Latimer-Cheung AE (2017). Sedentary behavior research network (SBRN) - terminology consensus project process and outcome. The international journal of behavioral nutrition and physical activity..

[6] Sedentary Behaviour Research Network. Letter to the editor: standardized use of the terms “sedentary” and “sedentary behaviours”. Applied physiology, nutrition, and metabolism = Physiologie appliquee, nutrition et metabolisme. 2012;37(3):540–2. 10.1139/h2012-024 22540258

[7] Sun F, Norman IJ, While AE (2013). Physical activity in older people: a systematic review. BMC Public Health.

[8] de Rezende LF, Rey-Lopez JP, Matsudo VK, do Carmo Luiz O (2014). Sedentary behaviour and health outcomes among older adults: a systematic review. BMC Public Health.

[9] Tremblay MS, Colley RC, Saunders TJ, Healy GN, Owen N (2010). Physiological and health implications of a sedentary lifestyle. Applied physiology, nutrition, and metabolism = Physiologie appliquee, nutrition et metabolisme.

[10] Sparling PB, Howard BJ, Dunstan DW, Owen N (2015). Recommendations for physical activity in older adults. Bmj..

[11] Sherrington C, Tiedemann A, Fairhall N, Close JC, Lord SR (2011). Exercise to prevent falls in older adults: an updated meta-analysis and best preactice recommendations. N S W Public Health Bull.

[12] Kraschnewski JL, Sciamanna CN, Ciccolo JT, Rovniak LS, Lehman EB, Candotti C, et al. Is exercise used as medicine? Association of meeting strength training guidelines and functional limitations among older US adults. Prev Med. 2014. 10.1016/j.ypmed.2014.05.012 PMC4162126 24878584

[13] Clarke T.C. NT, Schiller J.S.,. U.S. Department of Health and Human Services, Centers for Disease Control and Prevention. Early Release of Selected Estimates Based on Data From the 2016 National Health Interview Survey. Division of Health Interview Statistics, : National Center for Health Statistics,; 2017.

[14] Fleig L, McAllister MM, Chen P, Iverson J, Milne K, McKay HA (2016). Health behaviour change theory meets falls prevention: feasibility of a habit-based balance and strength exercise intervention for older adults. Psychol Sport Exerc.

[15] Arnautovska U, Fleig L, O'Callaghan F, Hamilton K (2017). A longitudinal investigation of older adults' physical activity: testing an integrated dual-process model. Psychol Health.

[16] Sheeran P (2002). Intention—behavior relations: a conceptual and empirical review. Eur Rev Soc Psychol.

[17] Yardley L, Donovan-Hall M, Francis K, Todd C (2007). Attitudes and beliefs that predict older people's intention to undertake strength and balance training. J Gerontol B Psychol Sci Soc Sci.

[18] Piercy KL, Troiano RP, Ballard RM, Carlson SA, Fulton JE, Galuska DA (2018). The physical activity guidelines for Americans. JAMA..

[19] Keller J, Fleig L, Hohl DH, Wiedemann AU, Burkert S, Luszczynska A (2017). Which characteristics of planning matter? Individual and dyadic physical activity plans and their effects on plan enactment. Soc Sci Med.

[20] Clemson L, Fiatarone Singh MA, Bundy A, Cumming RG, Manollaras K, O'Loughlin P (2012). Integration of balance and strength training into daily life activity to reduce rate of falls in older people (the LiFE study): randomised parallel trial. Bmj..

[21] Lally P, Gardner B (2013). Promoting habit formation. Health Psychol Rev.

[22] Warner LM, Schüz B, Knittle K, Ziegelmann JP, Wurm S (2011). Sources of perceived self-efficacy as predictors of physical activity in older adults. Applied Psychology: Health and Well-Being.

[23] Sniehotta FF, Scholz U, Schwarzer R (2005). Bridging the intention–behaviour gap: planning, self-efficacy, and action control in the adoption and maintenance of physical exercise. Psychol Health.

[24] Amagasa S, Machida M, Fukushima N, Kikuchi H, Takamiya T, Odagiri Y (2018). Is objectively measured light-intensity physical activity associated with health outcomes after adjustment for moderate-to-vigorous physical activity in adults? A systematic review. The international journal of behavioral nutrition and physical activity.

[25] Chastin SFM, De Craemer M, De Cocker K, Powell L, Van Cauwenberg J, Dall P (2018). How does light-intensity physical activity associate with adult cardiometabolic health and mortality?.

[26] Saint-Maurice PF, Troiano RP, Berrigan D, Kraus WE, Matthews CE. Volume of light versus moderate-to-vigorous physical activity: similar benefits for all-cause mortality? J Am Heart Assoc. 2018;7(7). 10.1161/JAHA.118.008815 PMC5907606 29610219

[27] Secretary of Health and Human Services. Physical Activity Guidelines Advisory Committee Report, 2008 Part A: executive summary. 2009 Feb. Report No.: 1753–4887 (Electronic) 0029–6643 (Linking) Contract No.: 2. 10.1111/j.1753-4887.2008.00136.x 19178654

[28] Fuzeki E, Engeroff T, Banzer W (2017). Health benefits of light-intensity physical activity: a systematic review of accelerometer data of the National Health and Nutrition Examination Survey (NHANES). Sports Med.

[29] Spring B., Moller A. C., Coons M. J. (2012). Multiple health behaviours: overview and implications. Journal of Public Health.

[30] Fleig L, Kerschreiter R, Schwarzer R, Pomp S, Lippke S (2014). ‘Sticking to a healthy diet is easier for me when I exercise regularly’: cognitive transfer between physical exercise and healthy nutrition. Psychol Health.

[31] Fleig L, Kuper C, Lippke S, Schwarzer R, Wiedemann AU (2015). Cross-behavior associations and multiple health behavior change: a longitudinal study on physical activity and fruit and vegetable intake. J Health Psychol.

[32] Fleig L, Lippke S, Pomp S, Schwarzer R (2011). Intervention effects of exercise self-regulation on physical exercise and eating fruits and vegetables: a longitudinal study in orthopedic and cardiac rehabilitation. Prev Med.

[33] Fleig L, Ngo J, Roman B, Ntzani E, Satta P, Warner LM (2015). Beyond single behaviour theory: adding cross-behaviour cognitions to the health action process approach. Br J Health Psychol.

[34] Nigg CR, Allegrante JP, Ory M (2002). Theory-comparison and multiple-behavior research: common themes advancing health behavior research. Health Educ Res.

[35] Ashe MC, Winters M, Hoppmann CA, Dawes MG, Gardiner PA, Giangregorio LM (2015). “Not just another walking program”: Everyday Activity Supports You (EASY) model - a randomized pilot study for a parallel randomized control study. Pilot and Feasibility Studies.

[36] Colley RC, Garriguet D, Janssen I, Craig CL, Clarke J, Tremblay MS (2011). Physical activity of Canadian adults: accelerometer results from the 2007 to 2009 Canadian health measures survey. Health Rep.

[37] Chang VC, Do MT (2015). Risk factors for falls among seniors: implications of gender. Am J Epidemiol.

[38] Lobo E, Marcos G, Santabárbara J, Salvador-Rosés H, Lobo-Escolar L, De la Cámara C (2017). Gender differences in the incidence of and risk factors for hip fracture: a 16-year longitudinal study in a southern European population. Maturitas..

[39] Michie S, Ashford S, Sniehotta FF, Dombrowski SU, Bishop A, French DP (2011). A refined taxonomy of behaviour change techniques to help people change their physical activity and healthy eating behaviours: the CALO-RE taxonomy. Psychol Health.

[40] Creswell JW, Clark VLP (2017). Designing and conducting mixed methods research: sage publications.

[41] Pant Pai N, Chiavegatti T, Vijh R, Karatzas N, Daher J, Smallwood M (2017). Measures and metrics for feasibility of proof-of-concept studies with human immunodeficiency virus rapid point-of-care technologies: the evidence and the framework. Point Care.

[42] Warburton DE, Jamnik VK, Bredin SS, Gledhill N (2011). The physical activity readiness questionnaire for everyone (PAR-Q+) and electronic physical activity readiness medical examination (ePARmed-X+). The Health & Fitness Journal of Canada.

[43] Rosenberger ME, Fulton JE, Buman MP, Troiano RP, Grandner MA, Buchner DM (2019). The 24-hour activity cycle: a new paradigm for physical activity. Med Sci Sports Exerc.

[44] Gibson W, Hunter KF, Camicioli R, Booth J, Skelton DA, Dumoulin C (2018). The association between lower urinary tract symptoms and falls: forming a theoretical model for a research agenda. Neurourol Urodyn.

[45] Noguchi N, Chan L, Cumming RG, Blyth FM, Naganathan V (2016). A systematic review of the association between lower urinary tract symptoms and falls, injuries, and fractures in community-dwelling older men. Aging Male.

[46] Hoffmann TC, Glasziou PP, Boutron I, Milne R, Perera R, Moher D (2014). Better reporting of interventions: template for intervention description and replication (TIDieR) checklist and guide. Bmj..

[47] Clemson L, Munro J, Fiatarone SM (2014). Lifestyle-integrated functional exercise (LiFE) program to prevent falls: trainer’s manual.

[48] Rosenberger ME, Fulton JE, Buman MP, Troiano RP, Grandner MA, Buchner DM, et al. The 24-hour activity cycle: a new paradigm for physical activity. Med Sci Sports Exerc. 2018. 10.1249/MSS.0000000000001811 PMC6377291 30339658

[49] Shoemaker SJ, Wolf MS, Brach C (2014). Development of the patient education materials assessment tool (PEMAT): a new measure of understandability and actionability for print and audiovisual patient information. Patient Educ Couns.

[50] Duckworth AL, Quinn PD (2009). Development and validation of the short grit scale (grit-s). J Pers Assess.

[51] Von Culin KR, Tsukayama E, Duckworth AL (2014). Unpacking grit: motivational correlates of perseverance and passion for long-term goals. J Posit Psychol.

[52] Strachan SM, Brawley LR, Spink K, Glazebrook K (2010). Older adults’ physically-active identity: relationships between social cognitions, physical activity and satisfaction with life. Psychol Sport Exerc.

[53] Anderson DF, Cychosz CM (1994). Development of an exercise identity scale. Percept Mot Skills.

[54] Gardner B, Abraham C, Lally P, de Bruijn GJ (2012). Towards parsimony in habit measurement: testing the convergent and predictive validity of an automaticity subscale of the self-report habit index. The international journal of behavioral nutrition and physical activity..

[55] Duckworth AL, Peterson C, Matthews MD, Kelly DR (2007). Grit: perseverance and passion for long-term goals. J Pers Soc Psychol.

[56] Rhodes RE, Kaushal N, Quinlan A (2016). Is physical activity a part of who I am? A review and meta-analysis of identity, schema and physical activity. Health Psychol Rev.

[57] Whitney JC, Lord SR, Close JC (2005). Streamlining assessment and intervention in a falls clinic using the timed up and go test and physiological profile assessments. Age Ageing.

[58] Thorne S, Kirkham SR, MacDonald-Emes J (1997). Interpretive description: a noncategorical qualitative alternative for developing nursing knowledge. Res Nurs Health.

[59] Sherrington C, Fairhall NJ, Wallbank GK, Tiedemann A, Michaleff ZA, Howard K (2019). Exercise for preventing falls in older people living in the community. Cochrane Database Syst Rev.

[60] Kelsey JL, Prill MM, Keegan TH, Tanner HE, Bernstein AL, Quesenberry CP (2005). Reducing the risk for distal forearm fracture: preserve bone mass, slow down, and don't fall!. Osteoporos Int.

[61] Mitchell RJ, Lord SR, Harvey LA, Close JC (2015). Obesity and falls in older people: mediating effects of disease, sedentary behavior, mood, pain and medication use. Arch Gerontol Geriatr.

[62] Anderson D, Cychosz C, Franke W (2001). Preliminary exercise identity scale (EIS): norms for three adult samples. Journal of Sport Behaviour.

[63] Prokop NW, Hrubeniuk TJ, Senechal M, Bouchard DR (2014). People who perceive themselves as active cannot identify the intensity recommended by the international physical activity guidelines. Open Access J Sports Med.

